# Male Fertility as a Proxy for Health

**DOI:** 10.3390/jcm13185559

**Published:** 2024-09-19

**Authors:** Calvin C. Zhao, Michael Scott, Michael L. Eisenberg

**Affiliations:** Department of Urology, Stanford University School of Medicine, Stanford, CA 94305, USA; cczhao@stanford.edu (C.C.Z.); micscott@stanford.edu (M.S.)

**Keywords:** male factor infertility, health outcomes

## Abstract

Male fertility is affected by a wide range of medical conditions that directly and indirectly affect spermatogenesis. As such, it can be useful as both an indicator of current health and a predictive factor for future health outcomes. Herein, we discuss the current literature regarding the association between male fertility and systemic health conditions and exposures. We review the connection between male fertility and genetics, medications, diet, and environmental pollutants, as well as its effects on future oncologic, cardiovascular, and autoimmune conditions. Understanding this interplay will allow more health care providers to engage in health counseling that will not only improve men’s reproductive outcomes but also their overall health.

## 1. Introduction

Approximately 15% of couples experience difficulty conceiving after a year of regular, unprotected sex, a condition known as infertility. Male infertility, responsible for 30–50% of these cases, affects approximately 7% of men worldwide, making it a public health issue [1,2,3]. With declining global sperm counts, physicians are increasingly likely to encounter patients with abnormal semen parameters [1].

Recent studies have indicated that male infertility could be a marker for broader health issues [4]. Infertile men often have more medical conditions compared to fertile men, and worse health is linked to poorer semen quality [5,6,7]. The etiology is uncertain, but investigators have suggested genetic, epigenetic, developmental, social, lifestyle, and exposures as possible etiologies [8,9]. Research is ongoing to better understand the link and hopefully offer counseling to men. Thus, men with abnormal semen analyses may benefit from comprehensive health assessments, including lifestyle and chronic disease management. Indeed, Shiraishi and colleagues demonstrated that infertile men who had controlled hypertension had significant increases in sperm counts [10].

As such, reproductive clinicians’ roles are evolving, with a focus on interdisciplinary care, thus improving both reproductive and overall health for men. This review summarizes the current literature on male fertility as a marker for current and future health.

## 2. Predictor of Future Health

### 2.1. Cancer

Several studies have suggested that men struggling with infertility are more prone to being diagnosed with testicular cancer [11,12,13]. Genetic instability and deficiencies in DNA repair within germ cells are thought to contribute to both spermatogenic failure and a heightened risk of cancer [14,15,16]. One major underlying cause is cryptorchidism, which causes the abnormal development or apoptosis of neonatal germ cells, leading to impaired and altered testicular development [17]. The prevalence of cryptorchidism remains high, approximately 1–3% in full-term newborns, and accounts for 10% of all testicular cancers [18]. If untreated, it leads to azoospermia in 13% of patients with unilateral cryptorchidism and 89% in those with bilateral cryptorchidism [19]. With surgical treatment, outcomes are improved; however, approximately 10% of infertile men still have a history of cryptorchidism and orchiopexy [20].

Testicular cancer also directly can impair spermatogenesis in a multitude of ways. Elevated serum alpha fetoprotein (AFP) or beta human chorionic gonadotropic (beta-HCG) can interfere with the hypothalamic–pituitary–gonadal (HPG) axis. The inflammatory response to cancer and local invasion can also disrupt testicular tissue through direct destruction, oxidative stress, and higher temperatures. Also, disruption of the blood–testis barrier in cancer can lead to the formation of anti-sperm antibodies. Chemotherapy and radiation, adjunctive treatments in many testicular cancers, have well-recognized gonadotoxic effects as well. Studies have shown that five-year azoospermia rates after chemotherapy can vary from 20 to 25%, depending on the regimen [21,22,23]. Lastly, retroperitoneal lymph node dissection, often performed in a salvage setting, has a high risk for anejaculation or retrograde ejaculation, estimated at approximately 10% when a nerve-sparing approach is used [24]. While treatment and outcomes for testicular cancer are highly variable, one study showed that the overall paternity success rates in patients undergoing surveillance, chemotherapy, and chemotherapy plus radiotherapy were 85%, 71%, and 67% respectively, even with the use of assisted reproductive technology [25].

Prostate cancer has also been linked to male infertility [26]. Research has indicated that men who undergo in vitro fertilization (IVF) have a higher likelihood of developing prostate cancer compared to those who conceive naturally. A systematic review and meta-analysis by Del Giudice et al. discovered that men with infertility are approximately twice as likely to develop testicular and prostate cancers, although the overall risk is still low, at approximately 1% [27].

Poor spermatogenesis may also increase the risk of developing other types of cancer. A study by Eisenberg et al. found that infertile men had a higher frequency of all tumors compared to fertile men, including non-Hodgkin lymphoma. Importantly, the risk was higher in men with azoospermia than in those with oligozoospermia [28].

Interestingly, the higher risk of cancer among infertile men also appears in close family members as well. A recent study by Ramsay et al. used the population databases in Utah to identify 786 subfertile men and their families [29]. Among the oligospermic men, they found significantly increased familial cancer risks in the following five subtypes: bone and joint, soft tissue, uterine, Hodgkin lymphoma, and thyroid cancer. They also found increased odds of pediatric cancer diagnoses [29]. These findings suggest that male infertility may be a marker for health risks in both the individual and the family, potentially due to common exposures or genetic factors.

### 2.2. Cardiometabolic Diseases

Male infertility is frequently associated with metabolic disorders such as diabetes mellitus and metabolic syndrome, both of which are closely tied to insulin resistance. A study by Glazer et al. compared 18,499 patients with male factor infertility to 21,017 controls from two enrollment periods of the Danish National IVF Registry, and they found that the men with infertility had a higher prevalence of diabetes (OR 1.57 and 1.41 for the first and second enrollment period, respectively), though the overall incidence was relatively low (1.6%) [30]. Another study on 2572 men showed that childless men had higher odds of developing metabolic syndrome (OR 1.22) and diabetes (OR 2.12) compared to fathers [31].

It has been hypothesized that the driving force behind both metabolic syndrome and infertility is oxidative stress, which leads to endothelial dysfunction. This activates leukocytes which releases inflammatory cytokines and pro-inflammatory markers, which leads to more oxidative stress [32,33]. Oxidative stress impacts spermatozoa due to DNA fragmentation, and it also causes membrane damage through lipid peroxidation [34]. In a study on 106 infertile men and 51 controls, measurements of seminal oxidation-reduction potential, which is a quantitative measurement of oxidative stress, were shown to be predictive of both infertility and poor semen quality [35].

Metabolic syndrome leads to cardiovascular disease, which has also been shown to have links to male infertility. Eisenberg et al. found that men with infertility were more likely to develop ischemic heart disease and diabetes, even after accounting for obesity, smoking, and sociodemographic factors [36]. These men were also more prone to prediabetes and high blood pressure [36,37]. Importantly, these conditions remained across different races/ethnicities. The Trøndelag Health Study showed similar results, with links to coronary heart disease and stroke [38]. In a Taiwanese population study, Chen et al. found that infertile men had higher incidences of cardiovascular diseases (CVDs) after 15 years [39]. Another study on over 137,000 men found that childless men had a higher risk of dying from CVD compared to men with two or more children, suggesting a link between infertility and increased CVD risk [40]. Thus, identifying male infertility as a possible early warning sign for cardiovascular disease could play a vital role in preventative healthcare, particularly since heart disease is the leading cause of death in the United States.

### 2.3. Autoimmune Disorders

Male infertility is also associated with autoimmune disorders. A Danish study conducted by Glazer et al., which involved 24,000 men, found that those experiencing male factor infertility had a greater likelihood of developing multiple sclerosis (OR, 1.61) [41]. Similarly, research by Brubaker et al. using the MarketScan insurance claims database (Merative L.P., Ann Arbor, MI) revealed that men diagnosed with infertility had elevated risks of autoimmune diseases, including psoriasis, systemic lupus erythematosus, Graves’ disease, thyroiditis, and multiple sclerosis, following a diagnosis of infertility [42]. While there certainly could be confounding hormonal, metabolic, or genetic factors at play, these findings underscore how infertility can be a harbinger of comorbid autoimmune conditions.

### 2.4. Hospitalization and Mortality

Given the higher risk of cancer, cardiovascular disease, metabolic alterations, and autoimmune disorders, it is perhaps not surprising that male factor infertility is linked to higher mortality [43]. A study on 43,000 men with infertility showed that semen parameters were positively correlated with longevity. Eisenberg et al. also found that poor semen quality was associated with increased mortality risk, even after accounting for baseline health [44]. These findings were verified in a United States insurance claims analysis, which showed a higher risk of all-cause mortality with increased severity of spermatogenesis impairment, especially in men with azoospermia [45]. In Denmark, impaired semen quality was shown to be associated with a higher mortality rate, independent of fatherhood status [46]. A systematic review and meta-analysis by the same authors showed consistent results, though the overall absolute risk of death was minimal [47].

In addition, impaired semen quality has also been associated with a higher risk of hospitalization [48]. Moreover, semen quality appears more predictive than education, obesity, and smoking when examining such risks [49]. Boeri and colleagues demonstrated acute declines in the health statuses of infertile men in the decade after evaluation, and these declines were even steeper for azoospermic men [50].

## 3. Manifestations of Current Health

### 3.1. Genetics

Nearly 10% of the entire male genome plays a role in reproduction, and an underlying genetic abnormality is identified in 15–30% of cases of male factor infertility [51,52]. Thus, an initial diagnosis of infertility may be a reflection of a wider genetic syndrome. For example, the decreased expression of single-nucleotide polymorphisms within DNA mismatch repair (MMR) mechanisms (e.g., MLH1, MSH5, and PMS2), which are essential for maintaining the integrity of cellular DNA during replication, have been found to be linked to azoospermia or severe oligozoospermia [53,54,55,56,57,58]. Similar MMR defects are also associated with increased risk of multiple malignancies including retinoblastoma, melanoma, and gastric, breast, and ovarian cancers [59,60]. They are also a feature of many genetic instability syndromes including Lynch Syndrome and Bloom Syndrome [61]. It is unsurprising, then, that infertility has been linked to many of these syndromes [59,62]. Though genomic testing is not routine, healthcare providers should be aware that the genetic abnormalities that underpin many infertility diagnoses can have far-reaching effects on other aspects of a patient’s health.

This relationship is exemplified in patients with mutations in the cystic fibrosis transmembrane receptor (CFTR) gene. Classically, these mutations manifest reproductively with the congenital unilateral or bilateral absence of the vas deferens (CBAVD) and obstructive azoospermia [63]. This relationship has been confirmed in mouse models, and whole exon sequencing of men with obstructive azoospermia has revealed previously unidentified mutations [64,65,66]. Many CFTR mutations are also associated with the clinical syndrome of cystic fibrosis, which can have severe health ramifications via pulmonary and endocrine dysfunction [67]. As such, screening for CFTR mutations is recommended for both patients and partners in couples where a man has obstructive azoospermia due to vasal agenesis or an idiopathic etiology [68].

Klinefelter syndrome (KS) is another genetic syndrome associated with reproductive and somatic manifestations. Although KS is the most common sex chromosomal anomaly in males, only 25–50% of patients are diagnosed in their lifetime [69,70]. Approximately 85% of men diagnosed with KS have a 47,XXY karyotype, and nearly all are azoospermic [69,71]. These patients usually exhibit hypergonadotropic hypogonadism due to primary testicular failure, often necessitating surgical sperm retrieval and assisted reproductive technologies for biological offspring.

Men with KS also face urological concerns including hypogonadism and sexual dysfunction, and they often require testosterone supplementation [72]. Beyond reproductive issues, KS patients are at higher risk for insulin resistance, diabetes, cardiovascular disease, thromboembolism, and dyslipidemia, and they have increased risks for cancers such as breast cancer and hematologic malignancies [73,74,75]. These systemic consequences of KS illustrate the importance of appropriate diagnoses as these patients often require multidisciplinary care for optimal management.

Y chromosome microdeletions, particularly in the azoospermia factor (AZF) region, are a known genetic cause of azoospermia and severe oligozoospermia [76]. The American Urological Association recommends screening for Y microdeletions in men with sperm concentrations below 5 million/mL [68]. In a study on 4000 infertile Portuguese men, Yq microdeletions were identified in 4.6% of the study participants, with the majority associated with azoospermia [77]. Of these microdeletions, 56.8% were in subregion AZFc, with AZFa (4.7%) and AZFb (4.0%) being less common. Other studies have reported incidences as high as 16.9%, with similar rates of AZFc mutations [78]. Identifying the specific deletion is crucial for clinical management and patient counseling as those with AZFa or AZFb deletions are typically advised to use donor sperm or consider adoption due to low sperm retrieval success rates [20]. However, men with AZFc deletions are eligible for testicular sperm extraction, with successful sperm retrieval rates of approximately 50% and live birth rates of approximately 25% [79]. Moreover, the transmission of Y chromosome microdeletions to sons must be discussed with patients.

Recent data have indicated that Yq microdeletions may impact overall health beyond reproduction. These microdeletions have been identified in the brain, stomach, and urinary tract, and copy number variations (CNVs) within AZFb and AZFc genes have been linked to neuropsychiatric disorders, such as bipolar disorder, major depressive disorder, and language impairment [80,81]. Additionally, an analysis of the UK Decipher database found that 30% of men with CNVs within the AZF genes had intellectual disorders or delayed development. Y microdeletions have also been associated with increased risk of testicular germ cell tumors, with Moreno-Mendoza et al. reporting a four-fold increased risk of testis tumors in men with certain AZFc microdeletions [82]. These findings emphasize the importance of screening for Yq microdeletions as part of routine infertility evaluations.

There is increasing interest in the role of epigenetics in male infertility, with studies suggesting an association between DNA methylation and cases of idiopathic infertility [83]. One gene frequently studied in this context is mesoderm-specific transcript, which is also associated with adipocyte differentiation and has been implicated in the development of obesity [84,85]. Alterations to DNA methylation in mesoderm-specific transcript have been linked to lower sperm concentrations and motility, morphologic changes, and recurrent pregnancy loss in couples [84,86,87]. Further research in epigenetic testing may enable more precise screening and treatment for both infertility and obesity-related diseases [88].

### 3.2. Medications and Substances

Both prescription medications and recreational substances have been associated with negative effects on male fertility.

5α-reductase inhibitors (5-ARIs) inhibit the conversion of testosterone to dihydrotestosterone and are commonly utilized for the treatment of benign prostatic hyperplasia and male pattern baldness, and a reduction in circulating DHT has been theorized to impact spermatogenesis and decrease total sperm count [89]. A randomized study by Amory et al. found that the daily use of dutasteride (0.5 mg) or finasteride (5 mg) for a year decreased sperm counts by approximately 30% and motility by 6–12%, though most impairments were not clinically significant and recovered after six months of discontinuation [90]. In contrast, a study by Overstreet et al. involving a lower dose of finasteride (1 mg) for 48 weeks, commonly used for alopecia, showed no impact on semen quality or hormone levels, suggesting a dose-dependent effect [91]. Additionally, a study on 4400 men undergoing fertility evaluation identified 27 who were taking finasteride as a possible etiology of male infertility. Upon discontinuation of the finasteride, these men experienced an 11.6-fold increase in sperm counts, though no changes in motility or morphology were noted [92]. Clinicians prescribing finasteride should consider advising patients about its potential negative impact on sperm quality.

Hypogonadism is known to have negative effects on male fertility, and the treatment of hypogonadism with exogenous testosterone is also an established cause of impaired spermatogenesis [93]. This, however, may not be clear to patients or even providers, as a past study demonstrated that 7 out of 59 (12%) urologists inappropriately prescribed exogenous testosterone as a treatment for male infertility [94]. Thus, it is important that there is appropriate patient counseling on the detrimental effects of testosterone replacement therapy on infertility, especially as an increasing number of men on testosterone have been undergoing infertility evaluations [93,95].

In fact, testosterone supplementation has been studied as a contraceptive, and it has been shown in multiple studies to often lead to azoospermia or severe oligozoospermia [96,97]. Fortunately, there is evidence that semen parameters can recover after the cessation of testosterone [95,98,99]. However, this recovery may be hampered by advanced age and the duration of testosterone use. One study on 66 men previously on testosterone demonstrated that for each additional year of age and testosterone therapy, the probability of recovery to a total motile count of 5 million was reduced by 1.7% and 3%, respectively. Only 70% regained spermatogenesis within 12 months after discontinuing testosterone, despite receiving treatment with human chorionic gonadotrophin or selective estrogen receptor modulators [100].

Another medication associated with male infertility is salicylazosulfapyridine (SASP), an anti-inflammatory medication used in the treatment of inflammatory bowel disease. SASP is composed of two molecules, sulfapyridine and 5-aminosalicylic acid (5-ASA), with sulfapyridine being more frequently associated with negative effects on fertility, though the exact mechanism is unclear [101,102,103]. A cross-sectional study on 21 men taking SASP found abnormal semen analyses in 18, of which 15 presented with oligozoospermia [104]. Abnormal semen was defined as having a low sperm density of less than 40 million per milliliter or poor mobility in 40% of sperm. In men with SASP-induced infertility, mesalazine is an effective alternative without known deleterious effects on fertility [105].

Hypertension is linked to poor semen quality, and its treatment can also affect semen parameters. A study found that men on beta-blockers for hypertension had significantly lower sperm concentrations, motility levels, and total counts. However, those using other antihypertensive medications, like calcium channel blockers or angiotensin receptor blockers, did not show changes in semen quality compared to the controls [106].

In addition to their effects on spermatogenesis, medications can impair fertility via impacts on ejaculation. α1-adrenergic receptor blockers are utilized in the treatment of BPH, with a known side effect of retrograde ejaculation. In a randomized study on 48 men, tamsulosin was associated with decreased ejaculatory volume in 90% of the men and anejaculation in 35% of the men [107]. Furthermore, tamsulosin was associated with a 13.8% reduction in motile sperm [108]. In contrast, alfuzosin did not cause anejaculation, possibly due to its selective α1-adrenergic activity [107]. Men starting on α-blockers should be counseled about its potential sexual side effects and fertility implications.

Several studies have suggested that various antidepressants may negatively impact semen parameters. A case series reported significant improvements in sperm concentrations and motility levels in two individuals with oligozoospermia after discontinuing selective serotonin reuptake inhibitors (SSRIs) [109]. Additionally, Tanrikut et al. found that 35 men taking paroxetine for 5 weeks had higher levels of sperm DNA fragmentation (30.3%) compared to baseline (13.8%) [109]. Furthermore, 74 men taking SSRIs showed decreased sperm motility levels (48.2% vs. 66.2%) and total sperm counts (61,200,000 vs. 186,200,000) compared to 44 healthy men [110]. Although the evidence is limited, it indicates that antidepressants may negatively affect male fertility.

Fertility can be impacted by recreational substances in addition to prescription medications. A 2017 meta-analysis found that daily alcohol use was linked to decreased semen volume and poor sperm morphology, while occasional alcohol use did not appear to be a risk factor [111]. Furthermore, a study on 776 infertile men by Bai et al. found that heavy alcohol consumption increased the risk of abnormal sperm concentrations, and Boeri et al. observed that heavy alcohol use resulted in worse semen quality compared to abstinence or moderate consumption in a study on 189 infertile men [112,113].

Similarly, heavy cigarette smoking has been associated with poor sperm quality. Compared to moderate smokers and non-smokers, heavy cigarette smokers were more likely to exhibit low sperm concentrations and poor motility levels [113]. This was further supported by a 2019 systematic review and meta-analysis showing that smoking had a negative effect on both sperm counts and morphologies [114]. Daily cigarette smoking was also found to be associated with sperm DNA fragmentation in a study on 160 men [115].

### 3.3. Diet

Good dietary habits have been consistently shown to improve semen quality. The Mediterranean diet, rich in vegetables, fruits, legumes, and whole grains, and low in saturated fats and meat, is linked with higher total sperm counts and lower risks of abnormal sperm concentrations and motility levels [116]. Similarly, diets rich in fruits, vegetables, poultry, seafood, and low-fat dairy are associated with improved semen parameters [117,118].

Fatty acids, particularly polyunsaturated fatty acids (PUFAs) like DHA, are essential for sperm cell membranes and fertilization processes. As PUFAs cannot be synthesized and must come from one’s diet, consuming omega-3 PUFA-rich fish oils has been linked to better sperm morphologies, concentrations, and motility levels [119,120]. Conversely, diets high in saturated and trans fats have been associated with reduced sperm counts and concentrations [121].

Isoflavones, commonly found in soy-derived products, have mild estrogenic effects due to their ability to bind to estrogen receptors, and they have been linked to smaller testicular sizes in animal models [122,123]. However, the impact of isoflavone intake on fertility in humans is unclear. A study on 609 men with male factor infertility found that increased isoflavone intake was associated with decreased sperm concentration and motility [124]. Conversely, a study on 48 men with abnormal semen parameters showed that dietary isoflavone supplementation was associated with higher sperm count and lower sperm DNA damage [125].

In addition to promoting longevity and reducing the risk of other chronic diseases, a healthier diet also positively affects male fertility. Men with infertility may benefit from dietary assessments and could require nutritional education or dietetic consultations.

### 3.4. Psychological Impacts

Chronic psychological stress can lead to systemic effects, disrupting the immune system, vasculature, nervous system, and, notably, the hypothalamic–pituitary–gonadal (HPG) axis, which is crucial for fertility. Disruptions in the HPG axis can alter growth hormone levels, decrease prolactin, and reduce testosterone [126]. While most studies focus on hormonal impacts, the effects on semen quality are not clear.

Zou et al. studied 384 men using a job content questionnaire and semen samples, finding that higher work stress significantly increased the risk of low sperm concentration (odds ratio 2.14) [127]. Interestingly, high social support mitigated this effect. Eskiocak et al. observed similar effects in 29 medical students, noting significant drops in sperm concentrations and motility levels during exam stress, which normalized three months later [128].

Bhongade et al. used the Hospital Anxiety and Depression Score (HADS) scale on 70 men within infertile couples, finding that 27% had high HADS scores. These men had lower sperm motility levels, morphologies, and counts, along with lower testosterone and higher gonadotropin levels [129]. A study on 1,215 Danish men using the Copenhagen Psychosocial Questionnaire also linked poor semen quality to higher stress levels, though no significant differences in reproductive hormone levels were found across the stress levels [130]. Overall, psychological stress appears to have a significant impact on male fertility, alongside other negative systemic effects.

In addition, there is increasing work exploring the physiological impacts of infertility in men, suggesting that there may be a bidirectional relationship between poor fertility and mental health. A 2012 review of the topic demonstrated that while men experience grief and have elevated anxiety during infertility treatment, they do not appear to have clinically significant symptoms of depression and anxiety. The authors attributed this to less visibility of men’s experiences of infertility and the societal pressures to compartmentalize distress [131]. Indeed, more recent survey data has suggested that men’s emotional needs are under-met when managing diagnoses of infertility [132]. It is, thus, important for providers to not only recognize existing psychosocial stress in men undergoing infertility treatment but also anticipate future distress.

### 3.5. Socioeconomic Impacts

Upon receiving infertility diagnoses, male patients must grapple not only with the medical challenges but also with the financial burden. Despite infertility being classified as a disease by both the American Society of Reproductive Medicine and the World Health Organization, only a few states in the United States mandate insurance coverage for treatments, including sperm retrieval and banking [133]. Infertility treatments are also costly; a study by Elliott et al. surveying 111 men seeking fertility care found that 47% experienced financial strain, with 64% incurring expenses over $15,000 [134]. Financial strain significantly impacts health outcomes and is associated with a higher risk of mortality compared to those without financial hardship [135]. It is crucial for healthcare providers to advocate for expanded fertility insurance coverage through legislative efforts on behalf of their patients.

### 3.6. Environment and Pollution

Environmental factors, particularly pollutants, significantly impact male fertility. Air pollution, containing carbon monoxide, sulfur dioxide, ozone, and particulate matter, is particularly concerning due to trace elements and endocrine disruptors [136]. Studies have linked air pollution to impaired semen parameters. For instance, Hammoud et al. found that decreased sperm motility was correlated with elevated air pollution levels, and Rubes et al. noted increased sperm DNA damage in high-pollution areas [137,138].

Bisphenol A (BPA), a common endocrine disruptor, is widespread in consumer products. Studies in animals and humans have shown that BPA exposure is associated with decreased sperm count, motility, and increased DNA damage [139,140,141,142]. Research by Li et al. in China and the LIFE study in the United States have linked higher BPA levels with impaired semen parameters [142]. However, these effects may be subtle as studies examining reproductive outcomes have not shown that BPA exposure was linked to time to pregnancy, live birth rate, or embryo quality in patients undergoing fertility treatment [142,143].

Perfluoroalkyl and polyfluoroalkyl substances (PFAS), used in various industries for their non-stick properties, do not degrade in the environment and are termed ‘forever chemicals’. Animal studies have shown that PFAS exposure can lower testosterone and sperm concentrations [144]. However, human studies have not consistently linked PFAS exposure to abnormal semen parameters, as evidenced by a meta-analysis by Bach et al., which found no clear associations [145].

Phthalates, synthetic chemical additives used in various consumer products, are prevalent in human exposure, primarily through contaminated food and beverages. A 2004 NHANES study found detectable phthalate metabolites in over 75% of urine samples in a representative United States population [146]. Phthalates disrupt hormones, increasing serum estradiol concentrations and decreasing testosterone synthesis [147,148]. Research has shown a dose–response relationship between urinary phthalate levels and decreased sperm motility and concentration [149]. Additionally, higher phthalate levels are correlated with lower testosterone, semen volume, and sperm count [150].

Pesticides can affect male fertility through skin contact or inhalation in occupational settings, like agriculture, and through dietary consumption. The pesticide 1,2-dibromo-3-chloropropane (DBCP) is notably linked to male infertility, and a 1977 study found that 14 out of 25 DBCP factory workers had azoospermia or oligozoospermia after more than three months of exposure [151]. Other pesticides, such as ethylene dibromide, malathion, and paraquat, also decrease sperm counts in exposed workers [152,153]. For the general population, the dietary intake of pesticide residues from fruits and vegetables can impact sperm quality. The Environment and Reproductive Health (EARTH) study found that high pesticide residue intake was associated with a 49% lower total sperm count and 32% lower normal morphology, while a low-to-moderate residue intake was linked to a higher total sperm count. However, pesticide residue levels did not affect reproductive hormone concentrations [154].

## 4. Conclusions

Managing male infertility goes beyond the goal of fathering children. It involves a thorough evaluation, including a detailed history, physical examination, and appropriate lab tests. This comprehensive approach can identify underlying genetic, lifestyle, and health factors with long-term implications, facilitating preventative counseling and interventions. Male fertility is increasingly seen as an indicator of overall male health. Ultimately, improvements in overall health can also manifest improvements in reproductive health.
